# Dialysis based-culture medium conditioning improved the generation of human induced pluripotent stem cell derived-liver organoid in a high cell density

**DOI:** 10.1038/s41598-022-25325-9

**Published:** 2022-12-01

**Authors:** Fuad Gandhi Torizal, Tia Utami, Qiao You Lau, Kousuke Inamura, Masaki Nishikawa, Yasuyuki Sakai

**Affiliations:** 1 Department of Bioengineering, Graduate School of Engineering, The University of Tokyo, Bunkyo-ku, Tokyo, Japan; 2 Department of Chemical Systems Engineering, Graduate School of Engineering, The University of Tokyo, Bunkyo-ku, Tokyo, Japan

**Keywords:** Pluripotent stem cells, Stem-cell differentiation

## Abstract

Human pluripotent stem cell-derived liver organoids (HLOs) have recently become a promising alternative for liver regenerative therapy. To realize this application, a large amount of human-induced pluripotent stem cells (hiPSCs) derived-liver cells are required for partial liver replacement during transplantation. This method requires stepwise induction using costly growth factors to direct the hiPSCs into the hepatic lineage. Therefore, we developed a simple dialysis-based medium conditioning that fully utilized growth factors accumulation to improve hepatic differentiation of hiPSCs at a high cell density. The results demonstrated that the dialysis culture system could accumulate the four essential growth factors required in each differentiation stage: activin A, bone morphogenetic protein 4 (BMP4), hepatocyte growth factor (HGF), and oncostatin M (OSM). As a result, this low lactate culture environment allowed high-density bipotential hepatic differentiation of up to 4.5 × 10^7^ cells/mL of human liver organoids (HLOs), consisting of hiPSC derived-hepatocyte like cells (HLCs) and cholangiocyte like-cells (CLCs). The differentiated HLOs presented a better or comparable hepatic marker and hepatobiliary physiology to the one that differentiated in suspension culture with routine daily medium replacement at a lower cell density. This simple miniaturized dialysis culture system demonstrated the feasibility of cost-effective high-density hepatic differentiation with minimum growth factor usage.

## Introduction

The liver is an essential organ that plays a vital role in various physiological functions. Partial or total liver dysfunction may cause life-threatening systemic physiological disturbances. Currently, orthotopic liver transplantation is the most effective and preferred therapy for liver failure. However, the shortage of liver donors is a major limitation of their therapeutic modalities. Human liver organoids (HLOs) consisting of hepatocyte-like cells (HLCs) and cholangiocyte like-cells (CLCs), which are differentiated from human-induced pluripotent stem cells (hiPSCs), may become a good source for personalized regenerative therapy for liver diseases^1–3^. Generally, 2 × 10^8^ liver cells per kg body weight are required to regenerate and restore the physiological function of liver failure or liver cirrhosis to ensure therapeutic efficacy^4^. To realize this translational application, a large-scale and cost-effective biomanufacturing process must be employed to improve the production of hiPSCs derived-liver cells^5^. Nutritional supply, waste metabolic byproduct removal, and mechanical-related stress are the key challenges that must be overcome in the current hiPSC culture. In addition, using growth factors caused by daily medium removal can lead to high production costs and inefficient differentiation processes^6–8^.

Dialysis culture system is a potential method that can be utilized to improve differentiation efficiency. This culture system was first employed to produce a monoclonal antibody from hybridoma cell lines with a high cell density^9,10^. Our previous study has shown their potential in the highly efficient production of undifferentiated hiPSCs by the accumulation of essential growth factors required for maintaining pluripotency and proliferation, while simultaneously maintain the medium conditioning at a high cell density^7,11^. Since the hepatic differentiation process requires a larger amount of these expensive factors, the cost reduction may be significantly enhanced using this dialysis device.

Here, we assessed the feasibility of hepatic differentiation of hiPSCs using a simple, miniaturized dialysis culture system. This system has successfully provided a low lactate environment and accumulates the primary growth factor in each step of differentiation, which is necessary to direct differentiation into a hepatic lineage at a high cell density. Furthermore, the proliferation was improved by adjusting the medium viscoelasticity with the inclusion of the gellan gum biopolymer FP001. These enhancements of culture conditions minimized the demand for expensive growth factor supplementation and improved the hepatic function of the resulting HLOs. Although this study was limited to hepatic differentiation, the possibility of cost reduction by completely utilizing endogenous and exogenous factors from high-density differentiation provides insight into the large-scale production of various hiPSC-derived organoids.

## Results

### Simple dialysis culture system and conventional medium replacement system

**Figure 1:**
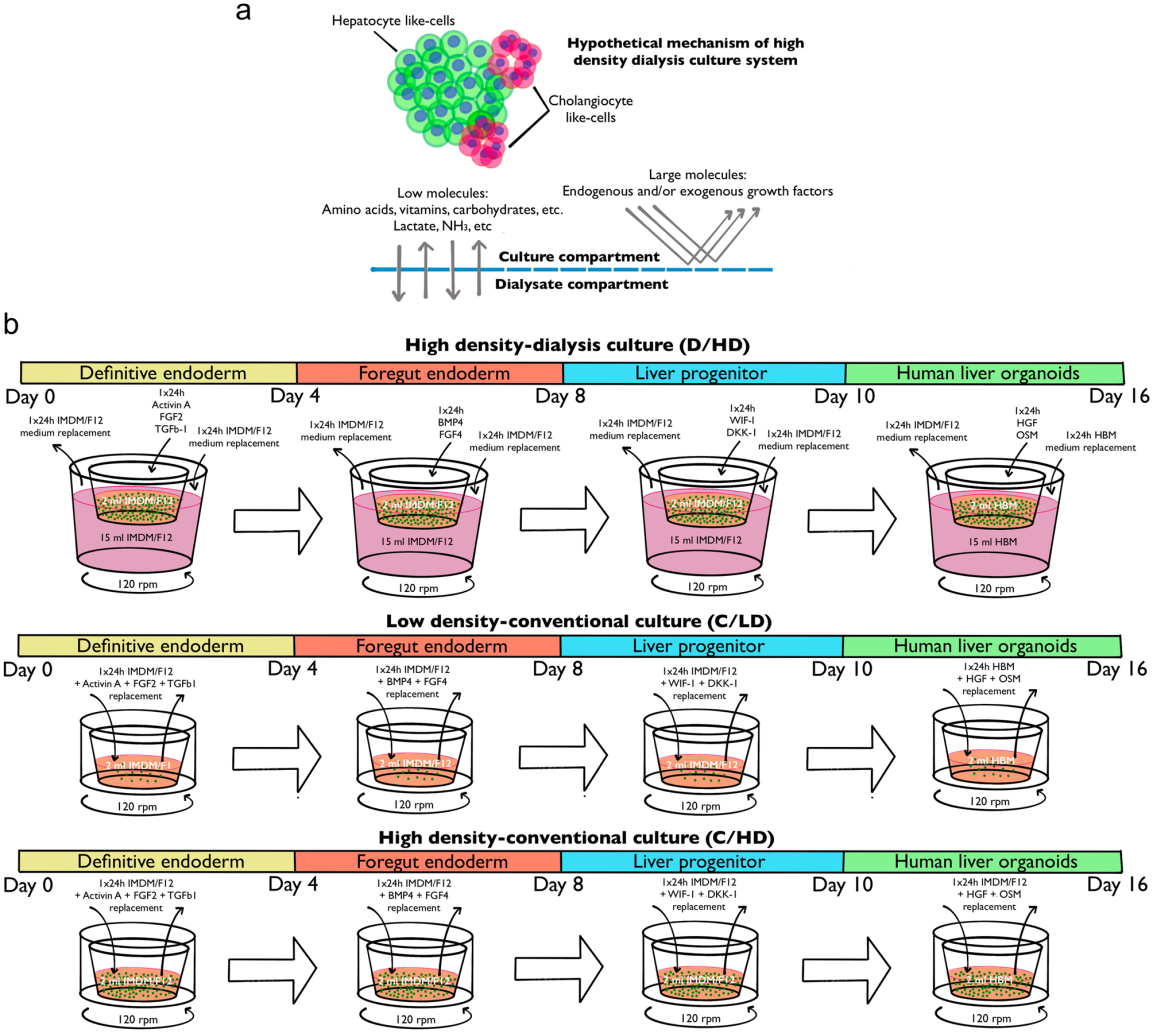
The experimental methodology used in this study. (**a**) The hypothetical mechanism of a high-density dialysis culture system utilizes the semipermeable dialysis membrane to accumulate the large molecule while maintaining culture condition by small molecule exchange. (**b**) The stepwise protocol of hepatic differentiation in the different culture settings and their medium replacement techniques.

To assess the differentiation efficiency, we performed stepwise hepatic differentiation in three configurations (Fig. 1a). In this hepatic differentiation study, we utilized the important growth factors by selectively accumulating them while maintain the culture conditioning by continuous exchange of low molecular weight nutrition and waste metabolic byproducts, enabling hepatic differentiation at high cell densities using a simple dialysis culture device (D/HD) (Fig. 1a,b). In D/HD, the dialysate medium was replaced daily, while growth factors were added to the culture compartment every 24 h without replacing the conditioned medium to accumulate the macromolecule growth factors. The exception was applied at the end of each stage; the culture medium and growth factors were completely replaced with the different formulated medium for the next stage. To evaluate the performance of this system, we included hepatic differentiation using a routine daily complete medium and growth factor replacement without dialysis to show the effect of conventional medium replacement on lower cell density (C/LD). For comparison, we added C/HD to represent the cellular defect that may occur when we forced high cell density differentiation using the same conventional medium replacement method (Fig. 1b).

### Improvement of HLOs generation in viscoelastic culture medium

Physical conditions such as excessive mechanical stress may considerably affect cell viability and differentiation efficiency in the hepatic lineage. Although a gentle dynamic culture system improved the hepatic differentiation^12,13^, the mechanical stress resulting from hydrodynamic mixing and aggregate collision in rotational culture may negatively interfere with cell viability and differentiation process^7^. A previous study by Vosough et al. revealed that excessive shear stress could reduce hepatic differentiation efficiency during culture in a stirred tank bioreactor^14^. Our previous study also showed that excessive hydrodynamic stress might reduce cell viability of the aggregates population^15^. In contrast, high-density culture may increase the risk of excessive agglomeration, resulting in a large fused aggregate prone to necrotic core formation^15,16^.

To address the problems, we added a gellan gum-based biopolymer FP001 to increase the viscoelasticity of the medium. This biomaterial exhibits viscous characteristics that resist the strain and shear stress and elastic characteristics making the medium flexibly adapt to the shape change and return to the original state during stress removal^17,18^. Because the rotational culture creates a continuous mechanical stress fluctuation, the viscoelastic medium can reduce the mechanical stress caused by dynamic suspension culture and aggregate collision^17^. The improvement of low mechanical stress in similar dialysis culture was tested in our previous study on hiPSC expansion; showed that the application of this biopolymer has effectively preventing excess agglomeration and protecting high-density hiPSC aggregates from mechanical stress-induced apoptosis and necrosis^7^. In this study, we confirmed that the inclusion of this biopolymer has shown its potential to improve the production of HLCs, indicated by higher HLOs production (Supplementary Fig. 1a) and better albumin expression (Supplementary Fig. 1b–d).

### Dense morphology and improved cellular proliferation

**Figure 2:**
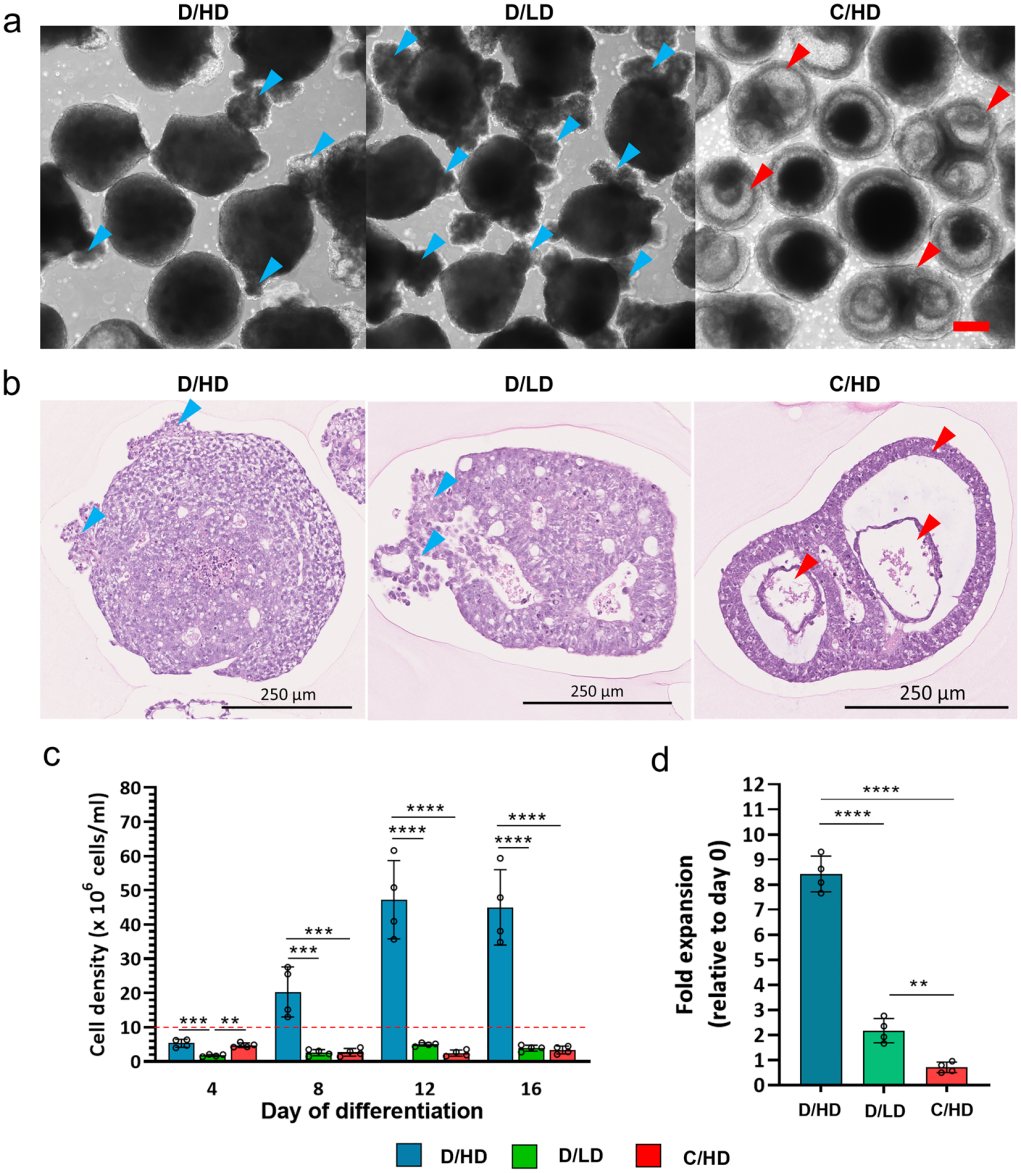
Morphology and growth rate of hiPSC-derived liver organoids (HLOs). (**a**) Morphological appearances of HLOs are generated from different cultural systems. The condensed cystic structure is depicted by blue arrows and cavitated cystic structure by red arrows. (**b**) The cross-sectioned histological morphology of the dense HLOs and complete cyst-like-HLOs. The condensed peripheral cystic structure showed by blue arrows and the cyst cavity showed by red arrows. (**c**) The medium conditioning in the dialysis system (D/HD) supported the HLOs differentiation in high density with (**d**) higher proliferation rate (n = 4 biologically independent experiments). The dotted line shows the minimum criteria of high-density mammalian cell culture, referring to Griffiths et al.

The HLOs from D/HD and C/LD represent similar morphological features that consist of a dense opaque core surrounded by small cystic structures in the peripheral area. Meanwhile, hiPSC aggregates differentiated in C/HD showed an enlarged cystic structure (Fig. 2a,b). Enhanced cellular proliferation was observed in the D/HD group compared to that in the C/LD and C/HD groups. The cells were proliferating until the foregut endoderm stage up to day eight at a high density (the high-density culture criteria were determined by ≥ 10^7^ cells/mL, according to Griffith et al.^19^), and stopped from day 12 (Fig. 2c). The reduction in this proliferation may be correlated with the loss of self-renewal capability during further cell fate specification and maturation^20^. This evidence suggests that the early stage of differentiation is an essential step in the production of a large number of cells, facilitated by continuous medium conditioning and growth factor accumulation in the dialysis culture system. In contrast, the cellular proliferation in both C/LD and C/HD was limited by the volume constraint of the 2 mL culture medium, resulting in a limited cell number, up to 3–6 × 10^6^ cells/mL (Fig. 2d).

### Medium conditioning enhances the culture environment during hepatic differentiation

**Figure 3:**
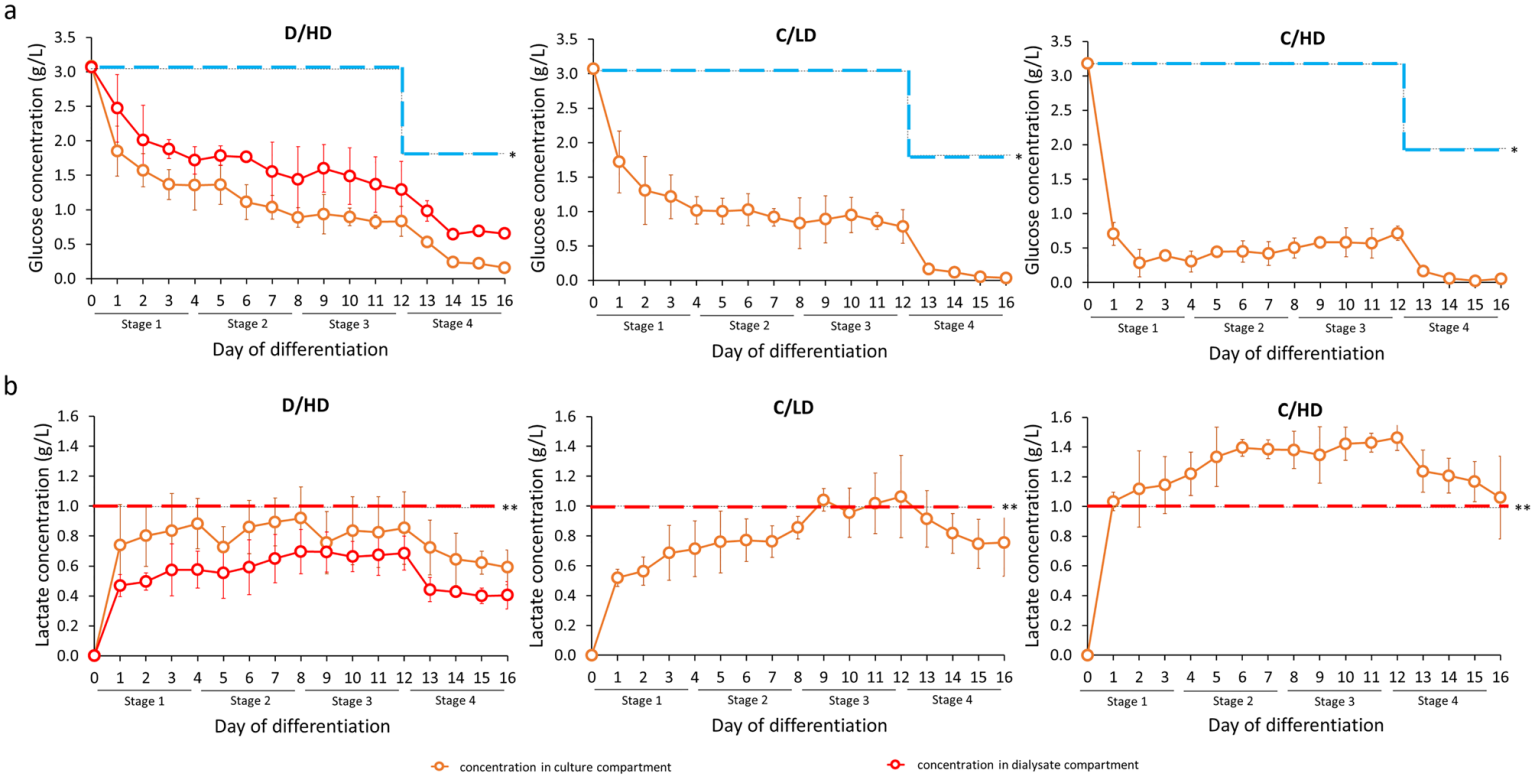
Glucose and lactate concentration in conditioned medium during differentiation. (**a**) The glucose concentration was sufficiently supplied by the dialysis culture system (D/HD). *The blue dotted line indicated the original glucose concentration in the culture medium. (**b**) The secreted lactate concentration was continuously removed and maintained below the critical limit of lactate accumulation that can impact viability. **This critical lactate concentration is based on the previous study by Horiguchi et al.^23^, indicated by the red dotted line. (n = 4 biologically independent experiments).

Glucose and lactate concentrations in the culture compartment were measured daily to evaluate the culture environment during high-density hepatic differentiation. Although glucose consumption was increased due to the proliferating cells, the dialysis support could accommodate the continuous supply of low-molecular-weight nutrition, such as glucose from the dialysate compartment (Fig. 3a). A different result was observed in the C/LD and C/HD groups that used a complete daily medium replacement. In C/LD, glucose was initially depleted due to increased glucose consumption, which correlated with increasing cell number. However, its daily concentration becomes similar on days 4–12 due to the inhibition of cell proliferation caused by the lactate that reaches the critical concentration. In contrast, the high inoculum cell density in C/HD caused a high lactate concentration that reduce cell number and high glucose consumption from the initial culture. Due to the lactate induced-cell death and decrease in glucose consumption, the remaining glucose after daily medium replacement was slightly increase up to day 12. In the last stage of differentiation, the glucose concentration in C/LD and C/HD was lower due to the low glucose formulation in the hepatocyte-basal medium (HBM). This low-glucose condition is necessary to adapt the HLOs toward a specific way of hepatic metabolism that utilizes galactose rather than glucose^21,22^.

The dialysis culture system also shows decent continuous lactate removal, so it can be maintained below the critical lactate concentration that may damage hiPSCs through acidification, as previously described by Horiguchi et al.^23^. In contrast, C/LD showed increased lactate accumulation above its critical concentration during the third stage of differentiation (Fig. 3b). This high lactate concentration may negatively affect proliferation^24^ and hepatic differentiation. The condition was more severe in C/HD, which experiences a high lactate environment during hepatic differentiation, resulting in morphological alteration into a low-proliferated-cystic-like structure. After the hepatoblast stage, the HLOs showed metabolic maturation towards HLOs on days 13–16. This phenomenon is indicated by the metabolic shift from glycolysis to oxidative phosphorylation during hepatic specification, which is more efficient in generating ATP and results in decreased lactate secretion^25,26^.

### Accumulation of the four essential factors in hepatic differentiation

One of the advantages of a dialysis culture system is its ability to prevent the loss of macromolecular components caused by complete medium replacement in conventional culture techniques. In our study, we added the same routine growth factor concentrations to all culture systems with different culture conditions related to lactate exposure. In C/LD, we provided the sufficient growth factor amount at lower cell concentrations as a control (the administration of growth factors per cell will be higher compared with D/HD and C/HD). We added C/HD to represent the defect that may occur when we forced differentiation at a high cell density with possible high lactate exposure and less exogenous growth factor availability per cell.

**Figure 4:**
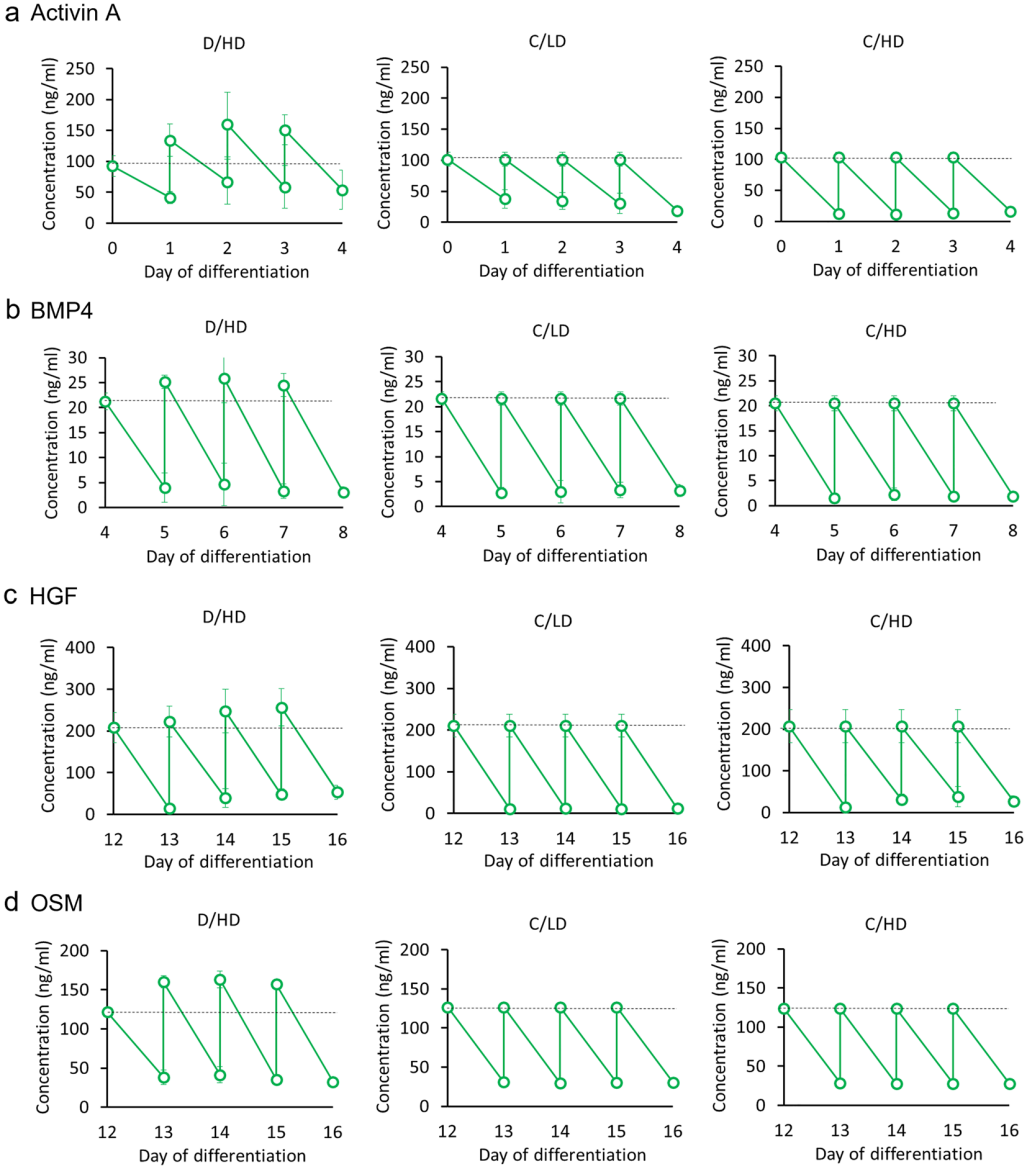
Growth factor accumulation for each culture configuration used in this study. The dialysis culture system (D/HD) successfully accumulated the four primary growth factors for hepatic differentiation, (**a**) activin A, (**b**) BMP4, (**c**) HGF, and (**d**) OSM contributed to hiPSCs derived liver organoids (HLOs) during their specific differentiation stage. (n = 4 biologically independent experiments). The dotted line indicates growth factor concentration in the differentiation medium.

The dialysis culture system (D/HD) can selectively accumulate large-molecular-weight growth factors essential for directed differentiation. To represent their accumulation, we selected four important growth factors that play a central role in hepatic differentiation: activin A, bone morphogenetic protein 4 (BMP-4), hepatocyte growth factor (HGF), and oncostatin M (OSM). These growth factors successfully accumulated in conditioned medium using D/HD, while their remaining concentrations were completely replenished during complete medium replacement in C/LD and C/HD. Since activin A and BMP-4 are endogenously secreted by hiPSCs^27,28^, their concentration can be maintained more efficiently during the definitive and foregut endodermal differentiation steps (Fig. 4a,b). HGF and OSM are important factors that direct hiPSC differentiation and maturation towards the hepatic lineage. The daily dose of these two components may not be sufficient for high-density differentiation because of their necessity to provide differentiation signals to a large number of cells. Similar to the conditions during liver development, these growth factors were also not natively secreted by pre-hepatic cells^29,30^. Thus, an additional autocrine effect cannot be achieved during this differentiation stage. In addition, HGF is highly sensitive to temperature-induced degradation over time^31,32^. Therefore, we investigated potential components that may improve the stabilization of these growth factors. The results demonstrated that the inclusion of fetal calf serum in the medium formulation significantly improved the stabilization of HGF and OSM in the culture medium by protecting them from degradation (Supplementary Fig. 2a). Subsequently, we performed an optimization experiment to obtain the optimum concentrations of HGF and OSM for high-density HLOs production (Supplementary Fig. 2b). Based on these optimizations, the HGF and OSM concentrations in the culture compartment were need to be increased to 200 ng mL^−1^ and 120 ng mL^−1^, respectively. Using these concentrations, HGF and OSM were sufficiently supplied and accumulated in the culture compartment (Fig. 4c,d).

### Continuous medium conditioning and growth factor accumulation supported hepatic differentiation

**Figure 5:**
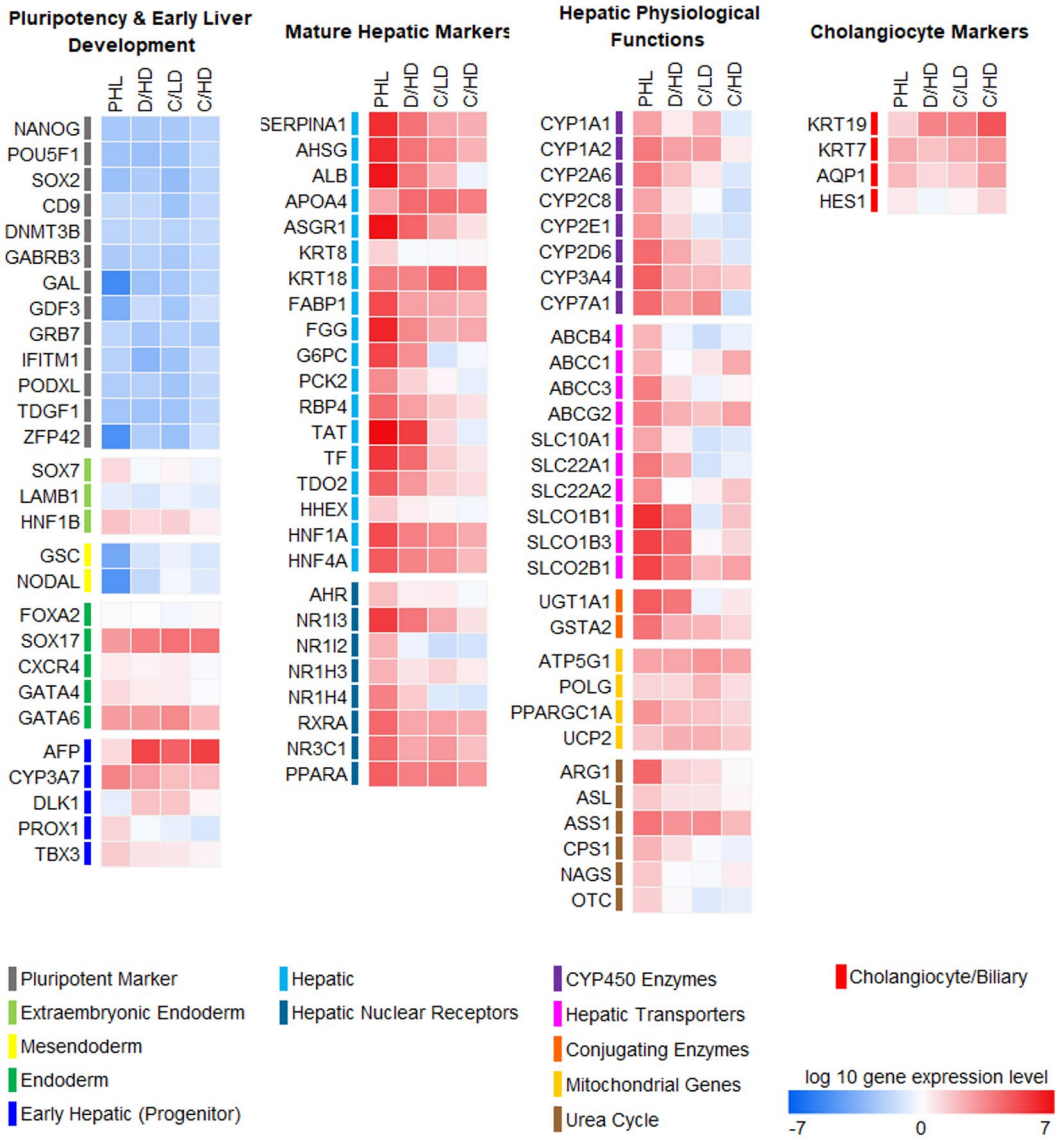
Comprehensive gene expression analysis of hiPSC-derived liver organoids (HLOs) and primary human liver (PHL). All expression levels demonstrated a relative expression level against the undifferentiated hiPSCs. The HLOs generated in D/HD conditioned medium showed a higher gene expression tendency of the hepatic-related markers than C/LD and C/HD. Additionally, the highest expression of cholangiocytes marker was detected in HLOs generated using C/HD (n = 6 biologically independent experiments).

We further analyzed the comprehensive gene expression profiles of fetal and adult liver-specific markers (Fig. 5). The results indicated that the HLOs generated in the conditioned medium using D/HD system showed a relatively higher expression of mature hepatic markers and their nuclear receptors. In this study, their physiological functions related to gene expressions, such as CYP450 enzymes, hepatic transporters, conjugating enzymes, mitochondrial genes, and urea cycle, were improved as well, compared with other culture systems. However, all resulting HLOs from hiPSCs still showed a relatively higher hepatic progenitor marker, such as alpha fetoprotein (AFP), and a lower hepatic marker compared with the primary human liver (PHL), which indicated that the maturation step still needs to be optimized.

**Figure 6:**
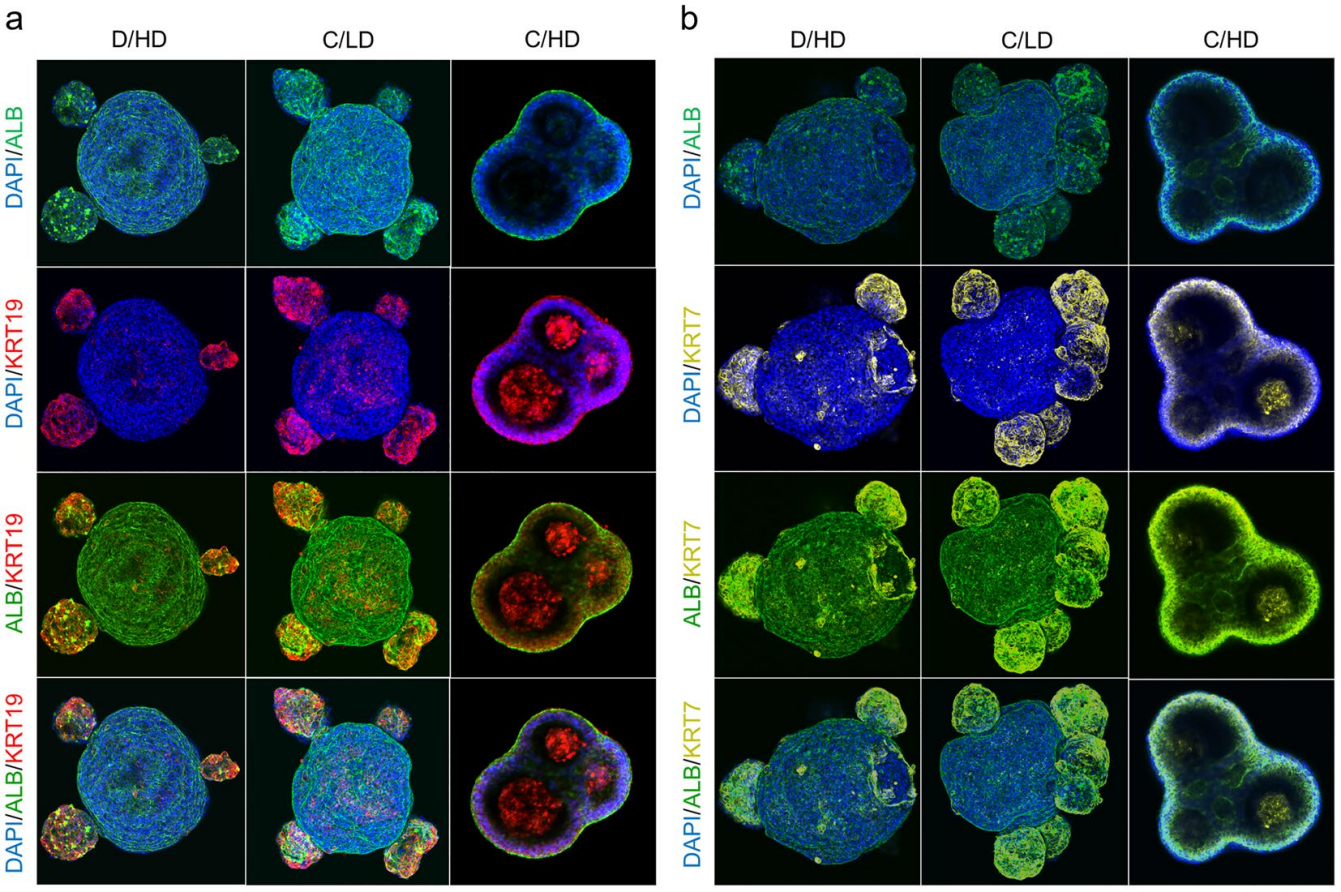
Characterization of differentiated human liver organoids (HLOs). The co-expression of cholangiocytes markers KRT19 (red) (**a**) and KRT7 (yellow) (**b**) were co-localized in the cystic region of the HLOs expressing ALB (green).

Liver progenitors often exhibit bipotential differentiation towards the hepatocyte and cholangiocyte lineage^33–39^. The differentiation tendency of these lineages can be affected by the culture environment. Notably, the high level of cholangiocyte markers in cystic-HLOs generated from C/HD has revealed that exposure to a high-lactate culture environment may increase the differentiation tendency into the cholangiocyte lineage. This tendency was confirmed by the upregulation of cholangiocyte marker gene expression (Fig. 5) and the localized expression of cytokeratin-19 (KRT19) and cytokeratin-7 (KRT7) among albumin (ALB)-positive cells localized in the cystic-like structure area, primarily in C/HD (Fig. 6a,b). Per another study conducted by Ramli et al.^35^ and Wu et al.^40^, this peripheral cystic structure is related to the development of the cholangiocyte lineage during hepatic differentiation. In our study, we observed that this cystic-like structure increased and grew persistently during exposure to a high-lactate culture environment in C/HD.

### Hepatic physiological functions of differentiated HLOs

**Figure 7:**
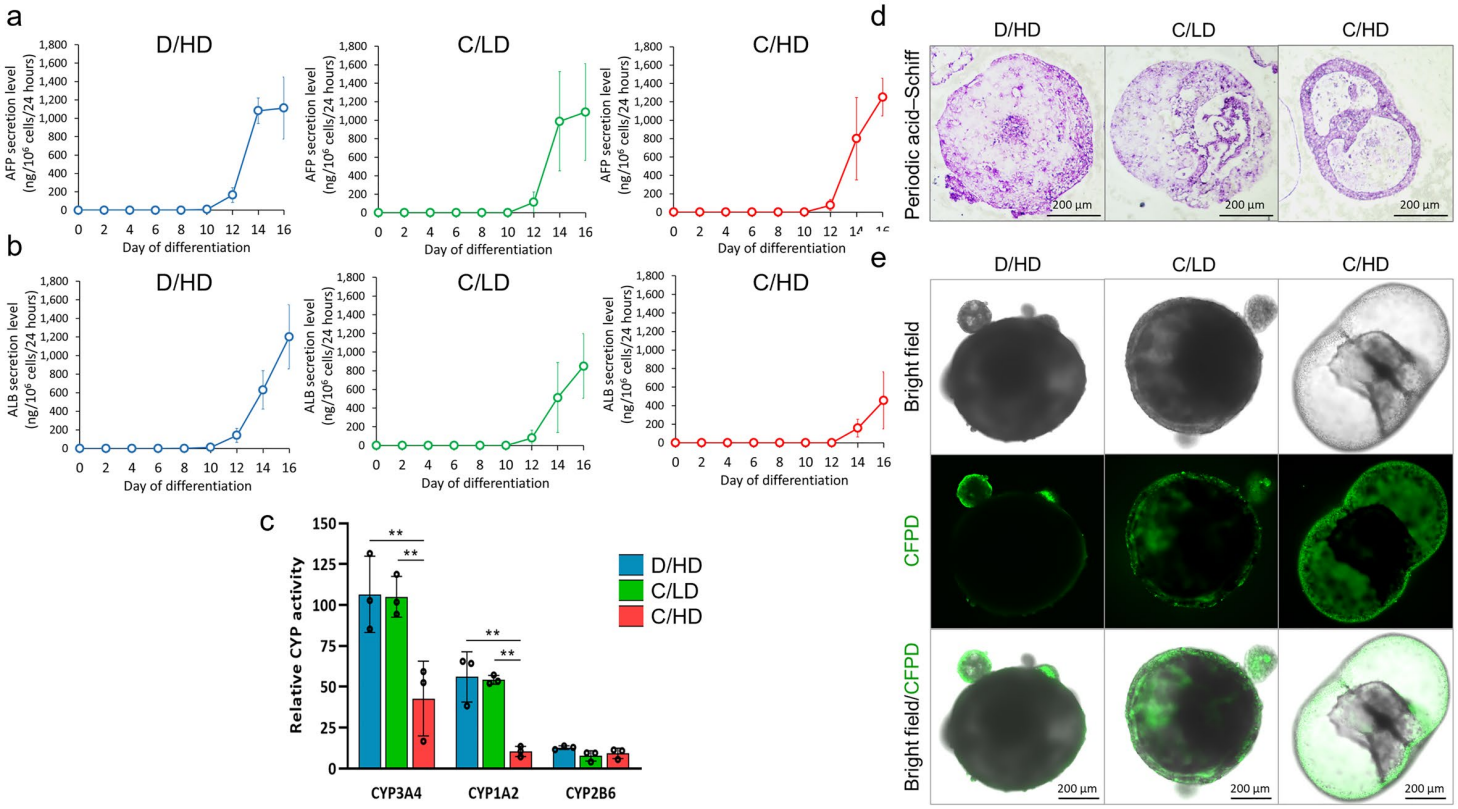
Physiological functions of hiPSC-derived liver organoids (HLOs). The HLOs differentiated in all systems showed a similar secretion level of liver embryonic protein marker AFP (**a**) and the one generated from D/HD showed slightly higher secretion of ALB (**b**) compared with the other HLOs in conventional medium replacement system (C/LD and C/HD) (n = 4 biologically independent experiments). After 24 h of incubation with a specific drug inducer, the HLOs differentiated in D/HD showed at least similar CYP450 activity with C/LD, and significantly higher than C/HD. All CYP450 value was normalized with its activity before drug induction (**c**), (n = 3 biologically independent experiments). All HLOs differentiated in all culture systems showed a glycogen storage capability (**d**). The visualization of active bile canaliculi in cystic structure counterparts is indicated by green CDFDA (**e**). Mean ± standard deviation is indicated in each graph. Statistical significance: **p < 0.01.

A higher albumin secretion was detected in the HLCs produced in the D/HD system compared with those in the C/LD system (Fig. 7a). The secretion levels of AFP were comparable among the HLOs generated in all culture systems at the end of differentiation (Fig. 7b), which may indicate immature liver development. This indicates that although the conditioned medium has no considerable advantage in reduction of AFP secretion, it could produce higher cell numbers compared to the HLOs generated in conventional culture.

To assess the metabolic activity of HLCs, we evaluated three CYP450 enzyme families. The HLCs produced in the D/HD conditioned medium indicated comparable CYP3A4 and CYP1A2 activity to those produced in C/LD, while C/HD revealed the lowest activity of these two enzymes. There was no difference in the activity of CYP2B6 among the culture systems (Fig. 7c). Additionally, the resulting HLOs that differentiated from all the culture groups showed a similar capability for glycogen conversion and storage (Fig. 7d).

Functional bile canaliculi were observed mainly in cystic HLOs produced from C/HD. The 5-carboxy-2,7-dichlorofluorescein diacetate (CDFDA) is accumulated in the cystic space of all HLOs, indicating the activity of the transporter protein of bile canaliculi, which consists of CLCs (Fig. 7e).

## Discussion

High production costs are the principal drawback in the translational application of hiPSC-based regenerative therapies for liver organs. Most differentiation processes involve stepwise induction by the combination of multiple exogenous growth factors, the most expensive components of the differentiation medium^38,41,42^. A simple miniaturized dialysis culture system is a potential method to maintain the exchange of small-molecule nutrition and waste metabolic products, supporting proliferation and differentiation, which enables a high-density volumetric yield^7^. This strategy successfully provided nutrition transfer and a low-lactate environment that supports cell growth by completely utilizing the accumulation of growth factors, which can be useful in the low-cost production of functional HLOs.

A decent culture environment was achieved in the production of HLOs from hiPSCs in a high cell density manner through a continuous supply of micromolecule nutrients, such as glucose, and waste metabolic products, such as lactate. Although the glucose concentration remained sufficiently high in all experimental groups, the accumulation of secreted lactate might become a critical problem by potentially disturbing proliferation and cellular viability through the lactate induced-acidification of the culture medium over time. Consistent with our previous study on high-density hiPSC expansion, the decreasing proliferation rate and hiPSC alteration were more affected by a high lactate concentration, rather than glucose starvation^7^. The dialysis operation relies on a micromolecule exchange between two compartments, which may reach concentration equilibrium at some point, depending on the dialysate volume. Therefore, to maintain the concentration equilibrium below the critical lactate concentration, we increased the volume of the basal medium to 15 mL. This volume was determined based on our previous expansion study of hiPSCs using a similar device^7^ that could maintain low lactate concentration produced by the high cell density for at least 24 h.

The conditioning of culture medium by dialysis system were able to maintain proliferation at an early stage and improved the functional maturation of the hepatic lineage. It was revealed that high exposure to lactate induces morphological alteration into a complete cystic-like structure with lineage specification tendency in cholangiocytes. Higher exposure to lactate may inhibit the proliferation of the outer layer of aggregates, resulting in smaller spheroid^7^. Based on our size-dependent hepatic differentiation study of hiPSC aggregates^43^, this smaller spheroid tends to activate Wnt signaling, which is a critical factor in driving hiPSCs toward a cystic form of cholangiocytes. In contrast, larger spheroids possess higher cadherin interactions that promote Wnt signaling inhibition, which is required to direct hiPSCs into hepatocytes in a dense aggregate form^43–45^. These findings provide important insights into the effect of excessive lactate exposure on lineage specification tendency during hepatic differentiation towards HLOs.

Our study demonstrated the successful accumulation of four essential growth factors during high-density hepatic differentiation in dialysis cultures. Activin A and BMP4 are the most important and costly growth factors in definitive endodermal and foregut endoderm differentiation, which is required at relatively high concentrations (100 ng/mL and 20 ng/mL, respectively)^46^. The inefficient utilization of these growth factors may cause failure in early lineage specification, resulting in non-endodermal cells that could not develop into the hepatic lineage^14^. Besides exogenous supplementation, these growth factors are endogenously produced by hiPSCs as paracrine factors to support their native homeostasis, which may reinforce their accumulation in the conditioned medium from culture compartment. In contrast, HGF and OSM, which are required for hepatic specification and maturation, are not natively produced by hiPSCs. These growth factors are produced by their different counterparts in the liver. HGF is produced by mesenchymal cells^29^ and exhibits a relatively short half-life in vivo^47–49^, whereas OSM is produced by hematopoietic cells during development^30^. Although their degradation can be prevented by FCS inclusion (Supplementary Fig. 2a), an additional amount is necessary to sufficiently supply these growth factors for HLOs at a very high cell density (Supplementary Fig. 2b).

Growth factor accumulation improves the differentiation of HLOs with mixed cells containing hepatocytes and cholangiocyte-like cells, which normally develop from bipotential hepatic progenitor cells (hepatoblasts)^50,51^. During liver organogenesis, hepatic specification factors such as HGF and OSM, which synergize with pre-hepatic inducers activin A and BMP4, play important roles in hepatocyte development^30,52,53^. Therefore, a sufficient number of these signaling molecules may significantly increase lineage specification toward hepatocytes rather than cholangiocytes during hepatic differentiation. The HLOs differentiated in dialysis culture displayed a higher composition of HLCs indicated by the higher levels of mature hepatocyte markers and their physiological functions, such as albumin secretion, drug metabolism, and glycogen storage (Fig. 7b–d). While the CLCs may exist in smaller amounts to provide a functional bile canaliculi ductular structure (Fig. 7e). This condition may correlate with the accumulation of endogenous and exogenous signals, which are known to promote maturation^54^.

This study revealed that this dialysis culture system could considerably reduce exogenous growth factor usage per cell unit production, which contributed to cost savings (Supplementary Fig. 3a,b). Our previous study showed that the accumulation profile of growth factors presented reproducible results among three undifferentiated hiPSC lines (including TkDN-4 M used in this study) expanded in a similar dialysis culture system^7^. Although we did not test hepatic differentiation with other hiPSC lines, according to this result, the accumulation profile may be expected to be similar across HLOs from different hiPSCs.

This simple culture platform can be utilized to optimize culture conditions using various parameters such as toxic cellular by-product accumulation from different cell densities, nutrition supply, and endogenous/exogenous growth factor kinetics. Although we successfully improved the HLOs production efficiency from the hiPSCs, several difficulties remain that need to be addressed to improve their efficacy for future translational applications. First, the resulting liver organoid presented a lower expression marker of mature hepatocyte counterparts compared with the primary human liver, which was also indicated by a high expression of fetal liver AFP. Although these profiles correspond with most hepatic differentiation studies from pluripotent stem cells (PSCs)^55^, fully mature organoids are essentially required for transplantation purposes. Several alternatives, such as a prolonged culture period with a maturation medium containing accumulated growth factor inducer, may be effective, but the physiological function often decreases over time during extended in vitro culture^56^. In addition, cell debris may accumulate and release harmful molecules. Second, scalable HLOs production is necessary for therapeutic purposes. For example, to regenerate one-tenth of the adult liver mass, approximately 10^9^ cells must be transplanted into patients^57^. Although multiple units of this simple dialysis platform can be employed in parallel, a larger dialysis culture system requires to be designed to simplify culture operations. Several studies have shown the possibility of performing hepatic differentiation of PSCs inside a hollow fiber-based dialysis^58,59^, but the complexity and difficulties in harvesting have also become a significant technical challenge that needs to be addressed. Therefore, we are currently attempting a scale-up method using a rotational culture with medium separation that perfused into a larger dialysis surface using an hollow fiber module.

In conclusion, the simple dialysis culture system could enhance the HLOs differentiation efficiency at a high cell density by facilitating a low lactate culture environment and proper nutrition supply that supported the accumulation of essential growth factors. Our study provides insights into feasible minimum growth factor utilization, which promotes a significant cost reduction during liver organoid differentiation.

## Methods

### Design of a simple dialysis culture system

The simple dialysis-culture system consists of upper culture compartment and lower dialysate compartment. A mesh bottom-cell strainer (PluriSelect, Leipzig, Germany) was modified by cutting and replacing the bottom mesh layer with a 12-kDa MWCO Spectra/Por 4 dialysis membrane (Spectrum Chemical, New Brunswick, NJ, USA). The dialysis membrane was affixed to the bottom of the strainer using an alkyl-α-cyanoacrylate-based surgical-grade tissue adhesive (Aron Alpha A; Daiichi Sankyo, Japan). The upper dialysis culture compartment inserts were then placed in 6-deep well plates (Corning, NY, USA). To represent the control condition, the cell strainer was directly affixed to the bottom surface of six-well-plates (Iwaki, Tokyo, Japan) using Aron Alpha A tissue adhesive (Daiichi Sankyo). All the devices were sterilized using an ethylene oxide gas sterilizer before use.

### Monolayer hiPSC cultures

The TkDN-4M hiPSC line was provided by the Stem Cell Bank, Center for Stem Cell Biology and Regenerative Medicine, University of Tokyo (Tokyo, Japan)^55^. The cells were maintained in vitronectin-coated tissue culture dishes using an Essential 8 (E8) culture medium (Thermo Fisher Scientific, Waltham, MA, USA).

### Hepatic differentiation of hiPSCs in high-density suspension cultures

Human induced pluripotent stem cells (hiPSCs) was harvested from a monolayer culture and dissociated into single-cell suspensions by passing through a 40 µm strainer (Corning, USA). hiPSC aggregates were formed by inoculating 2 × 10^6^ single-cell suspensions per well in six-well plates for 24 h in mTeSR1 medium using a 90-rpm rotary shaker. The differentiation protocols were adapted from a previous study by Pettinato et al.^46^ with several modifications. The differentiation components were divided into two groups in different compartment to generate high-density HLOs using a dialysis culture (Table 1). The low-molecular-weight (LMW) components consisted of a basal medium with antioxidants and antibiotics. The high molecular weight components (HMW) component consisted of growth factors differentiation cocktails, fetal calf serum, and a gellan gum-based-biopolymer that prevent excess agglomeration and reduced shear stress. A total of 8 × 10^6^ cell aggregates were collected from four wells of a six-well-plate in a 10 mL tube. The mTeSR1 medium was replaced with 2 mL of differentiation medium containing LMW and HMW components, resulting in a total cell density of 4 × 10^6^ cells/mL. The cell suspension was placed in the upper culture compartment. To continuously provide nutrition and dilute lactate, 15 mL of the LMW component was placed in the lower dialysate compartment. The dialysis culture was performed at a rotation speed of 120 rpm.

**Table 1:** The composition of low- and high-molecular-weight components in this study.

Differentiation stage	Low molecular weight component	High molecular weight components	
		Growth factors	Another material
Definitive endoderm	IMDM/F12 (1:1)	5% fetal calf serum (FCS)	2% FP001
	55 µM Monothioglycerol	1% Insulin-Transferrin-Selenium (ITS)	
	1% Penicillin–streptomycin	10 ng mL^−1^ FGF2	
		100 ng mL^−1^ Activin A	
		10 ng mL^−1^ TGFβ-1	
Foregut endoderm	IMDM/F12 (1:1)	5% fetal calf serum (FCS)	2% FP001
	55 µM Monothioglycerol	1% Insulin-Transferrin-Selenium (ITS)	
	1% Penicillin–streptomycin	10 ng mL^−1^ FGF4	
		20 ng mL^−1^ BMP4	
Pre-hepatic specification	IMDM/F12 (1:1)	5% fetal calf serum (FCS)	2% FP001
	55 µM Monothioglycerol	1% Insulin-Transferrin-Selenium (ITS)	
	1% Penicillin–streptomycin	1 µg mL^−1^ WIF-1	
		0.1 µg mL^−1^ DKK-1	
Hepatic lineage	HBM	5% fetal calf serum (FCS)	2% FP001
	55 µM Monothioglycerol	1% Insulin-Transferrin-Selenium (ITS)	
	1% Penicillin–streptomycin	200 ng mL^−1^ HGF	
		120 ng mL^−1^ OSM	

The daily medium replacement was performed by replacing 25 mL of the LMW medium in the dialysate compartment with fresh LMW medium, while a daily dose of HMW growth factors was added to the culture compartment only, without removing the previous LMW component, as described in Fig. 1. There is an exception at the end of each differentiation stage (Day 5, 9, and 13). The medium in the culture and dialysate compartment was completely replaced with the new differentiation induction medium formulation, followed by the previous dialysis medium replacement method.

### Morphological observation of liver organoids

The aggregate population were moved to a 60 mm culture dish (Iwaki, Tokyo, Japan) and morphological images were obtained using an Olympus IX83 light microscope (Olympus, Tokyo, Japan).

### Cell counting

HiPSCs derived-liver organoids (HLOs) were isolated by centrifugation at 1000 rpm for 3 min. The supernatant was carefully removed from the tubes. The aggregates were dissociated by adding 1–2 mL of Accutase solution (Thermo Fisher Scientific) and placed in a six-well-plate. The dissociation process was performed under 60 rpm rotation for 10–25 min at 37 °C, followed by gentle homogenization using a pipette to obtain the single cells. The cell solution was diluted in Trypan Blue (Thermo Fisher Scientific) and counted using a hemocytometer (SLGC, Yokohama, Japan).

### Hematoxylin and eosin staining of cross-sectioned HLOs

The HLOs were fixed using 4% paraformaldehyde (Wako Pure Chemical) overnight, followed by incubation with 30% PBS-sucrose solution (Wako Pure Chemical) for 24 h at 4 °C. The sucrose solution was removed. The aggregates were embedded with Tissue-Tek OCT compound (Sakura, Alphen aan den Rijn, The Netherlands) in a cryo-mold at − 20 °C until hardening. Thin sections (10 µm) were obtained using a cryostat and mounted on glass slides for hematoxylin–eosin staining. The samples were observed under an Olympus IX83 light microscope (Olympus).

### Measurement of glucose and lactate concentrations

Glucose and lactate concentrations during hepatic differentiation were measured by collecting 50-µL of medium samples every 24 h using an OSI BF-48AS Bioanalyzer (Oji Scientific Instrument, Hyogo, Japan).

### Protein levels measurement by enzyme-linked immunosorbent assay (ELISA)

To measure growth factor accumulation during differentiation, such as activin A, BMP4, HGF, and OSM, 100 µL of culture medium was directly isolated from the culture compartment every 24 h.

To measure the secretion levels of AFP and ALB, approximately 50 aggregates were isolated from the high-density culture, washed twice with PBS, and moved into six-well-plates. Aggregates were cultured for 24 h in the differentiation medium. The concentration was normalized to the number of cells that dissociated from the aggregates to measure the secretion levels of ALB and AFP.

The activin A, BMP4, OSM, AFP, and ALB concentrations were measured using the Duo Set ELISA Kit (R&D Biosystems, Minnesota, USA). HGF was measured using an HGF Quantikine ELISA kit (R&D Biosystems, Minnesota, USA), following the manufacturer’s instructions. A 100-µL solution of diluted capture antibody per well of 96-well enzyme-linked immunosorbent assay (ELISA) plates was immobilized in each well and incubated overnight at 37 °C. The plate was then washed with 200 µL of wash buffer, followed by incubation with 300 µL of blocking buffer (reagent diluent) for 1 h at 25 °C.

Standard protein solution (100 µL) or each medium sample was diluted in a reagent diluent and plated in each well, followed by incubation for 2 h at 25 °C. The plates were washed with wash buffer and incubated with 100 µL/well of the diluted detection antibody (R&D Biosystems) for 2 h at 25 °C. Subsequently, 100 µL of streptavidin-horseradish peroxidase (HRP) solution (R&D Biosystems) was added and incubated for 20–39 min at 25 °C. The color reaction was generated by washing the plates with wash buffer and adding 100 µL of the substrate solution to each well. The samples were incubated for 20 min at 25 °C in dark places. A stop solution (50 µL/well) was then added to stop the color reaction. The fluorescence intensity was measured using a Wallace Arvo SX 1420 multilabel counter (PerkinElmer).

### Quantitative reverse-transcription PCR (qRT-PCR)

RNA was isolated using TRIzol reagent (Life Technologies). cDNA was synthesized using the ReverTra Ace master mix (Toyobo, Osaka, Japan). qPCR analysis was performed using the Thunderbird SYBR qPCR mix (Toyobo) according to the manufacturer’s instructions. Gene amplification was performed using a StepOnePlus qPCR kit (Thermo Fisher Scientific). The primer sequences used in this analysis are listed in Supplementary Table 1.

### Comprehensive gene expression analysis

Total RNA was isolated using TRIzol reagent (Life Technologies) and reverse-transcribed using ReverTra Ace master mix (Toyobo, Osaka, Japan), followed by comprehensive qPCR analysis using Primer Array Hepatic Differentiation Kit (Takara Bio, Shiga, Japan) and KOD qPCR master mix (Toyobo) according to the manufacturer’s instructions. Gene amplification was performed using a StepOnePlus kit (Thermo Fisher Scientific, Massachusetts, USA). Comprehensive gene expression analysis was performed using the PrimerArray Analysis Tool for Hepatic Differentiation (Takara Bio, Japan).

### Immunohistochemistry

Approximately 30 HLOs were collected in microtubes and fixed in 4% paraformaldehyde for 12 h at 4 °C. The aggregates were then permeabilized with 1% Triton X (Thermo Fisher Scientific, Waltham, MA, USA) in PBS for 1 h. This was followed by additional incubation using a gelatin blocking buffer solution containing 1% PBS-Tween (Nacalai Tesque, Kyoto, Japan) for 1 h at room temperature by laying the tube horizontally in an orbital shaker. The protein targets were detected by incubation with 1 µg/mL primary antibodies for 3–4 days, followed by washing with PBS and incubation with 1 µg/mL secondary antibodies for 12–24 h at 4 °C in a rotary shaker. The aggregates were incubated with 1:1000 nuclear staining with 4′,6-diamidino-2-phenylindole (DAPI) (Dojindo, Kumamoto, Japan) for 30–40 min before observation. The antibodies used in this study are listed in Supplementary Table 2. Fluorescence imaging of the protein target was performed using an FV1200 confocal microscope (Olympus, Tokyo, Japan).

### CYP450 activity assays

Following the manufacturer's protocol, cytochrome P450 enzyme activity was measured using a P450-Glo Assay Kit (Promega, Madison, WI, USA). Approximately 100 aggregates were isolated from each group of culture systems at the end of the hepatic differentiation. For the CYP3A4 assay, the aggregates were incubated in an hepatocyte culture medium (HCM) with BulletKit supplementation and 20 μM rifampicin solution (Sigma-Aldrich) for 48 h. CYP1A2 activity was evaluated by incubating the aggregates in the HCM Hepatocyte Culture Medium with BulletKit containing 50 μM omeprazole solution (Sigma-Aldrich) for 48 h. For the CYP2B6 activity assay, the aggregates were incubated with the HCM with BulletKit supplement containing 1000 μM phenobarbital solution (Sigma-Aldrich, USA) for 48 h. The CYP activity value was normalized to the number of cells tested. The activity of CYP2B6 was tested using P450-Glo CYP2B6 (Promega, Madison, WI, USA), CYP3A4 using P450-Glo CYP3A4 (Promega), and CYP1A2 using P450-Glo CYP1A2 (Promega).

### Periodic acid-Schiff staining

Periodic acid Schiff staining was performed for thin sectioned aggregates using a periodic acid Schiff staining (PAS) staining system kit (Sigma-Aldrich, USA) according to the manufacturer’s instructions. The slide was immersed in the periodic acid solution for 5 min at room temperature followed by rinsing thrice with distilled water. The slides were then incubated with Schiff’s reagent for 15 min at room temperature, followed by washing them in tap water for 5 min. The counterstain was followed by 90-s incubation with hematoxylin solution (Gill no. 3), followed by rinsing in tap water. Xylene-based mounting solutions were applied to the slides, which were observed using an Olympus IX83 light microscope (Olympus).

### Visualization of bile canaliculi formation

Approximately 30 resulting HLOs were used for visualization of bile canaliculi. The organoids were incubated in HCM containing 1 µmol/L 5-(and-6)carboxy-2`,7`-dichlorofluoroscein diacetate (CDFDA) at 37 °C for 20–30 min. Subsequently, the HLOs were washed twice with HCM without CDFDA and observed using an FV1200 laser confocal microscope (Olympus, Tokyo, Japan).

### Statistical analysis

Statistical analysis was performed using the GraphPad Prism software (v.9.1.1; GraphPad Software, San Diego, CA, USA). Statistical significance was determined using a one-way analysis of variance followed by Tukey's multiple comparison test. Statistical significance was set at p < 0.05.

## Supplementary Information

Supplementary Information.

## Data Availability

The datasets generated and/or analysed during the current study are available in the Open Science Network (OSF) public repository, 10.17605/OSF.IO/JBDNT.
