# Author Correction: Dialysis based-culture medium conditioning improved the generation of human induced pluripotent stem cell derived-liver organoid in a high cell density

**DOI:** 10.1038/s41598-023-27875-y

**Published:** 2023-01-13

**Authors:** Fuad Gandhi Torizal, Tia Utami, Qiao You Lau, Kousuke Inamura, Masaki Nishikawa, Yasuyuki Sakai

**Affiliations:** 1 Department of Bioengineering, Graduate School of Engineering, The University of Tokyo, Bunkyo-ku, Tokyo, Japan; 2 Department of Chemical Systems Engineering, Graduate School of Engineering, The University of Tokyo, Bunkyo-ku, Tokyo, Japan

Correction to: *Scientific Reports* 10.1038/s41598-022-25325-9, published online 01 December 2022

The original version of this Article contained an error in the spelling of the author Qiao You Lau which was incorrectly given as Qiau You Lau.

The original Article has been corrected.

