# Supplementary material for: Dialysis based-culture medium conditioning improved the generation of human induced pluripotent stem cell derived-liver organoid in a high cell density
Source: Sci Rep. 2022 Dec 1;12:20774. doi: 10.1038/s41598-022-25325-9 (PMC9715714; doi:10.1038/s41598-022-25325-9)
Supplement: Supplementary file 1 — Supplementary Information. [file 41598_2022_25325_MOESM1_ESM.pdf]

Supplementary figure 1. Comparison between hepatic differentiation in monolayer (2D), suspension culture (3D), and suspension culture with viscoelastic gellan gum biopolymer FP001 (3D + GG). The exclusion of viscoelastic biopolymer FP001 in culture medium was relatively increase the cell production (a); relatively similar CYP3A4 (b), and significantly higher ALB (c). All hiPSCs differentiated in 3D showed a significantly lower cholangiocyte marker CK19, compared to the one differentiated in monolayer (d). (The data obtained from at least 3 biologically independent experiments; n 2D = 3; n 3D = 6; and n 3D+GG = 6). Statistical significance: *p < 0.05; ****p < 0.0001.

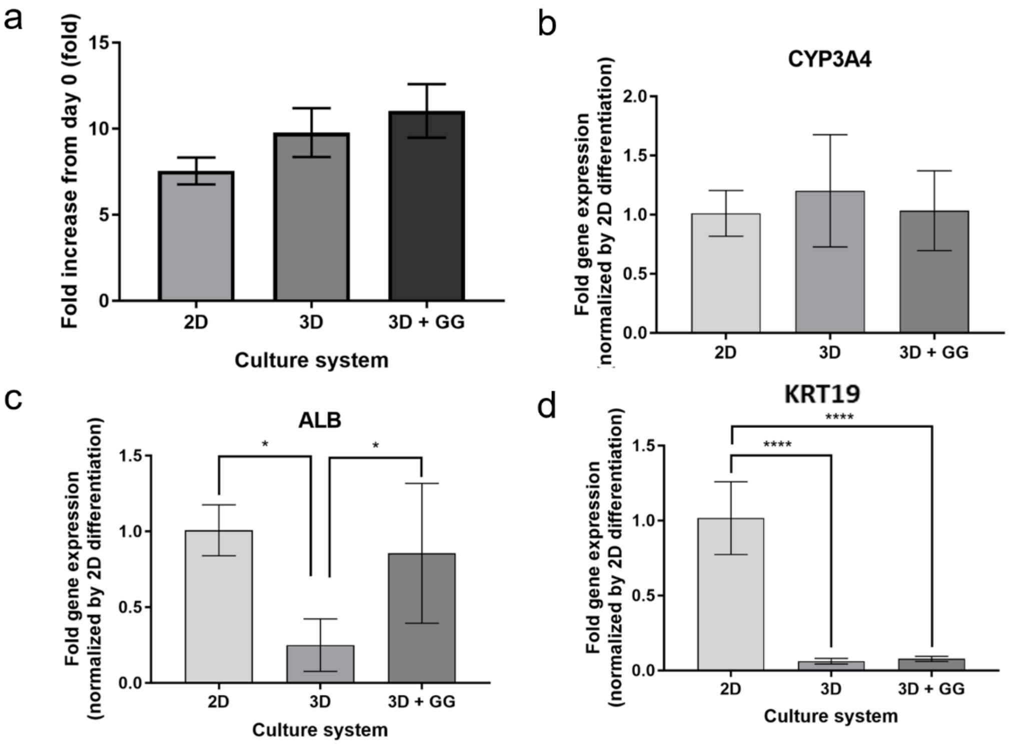

Supplementary figure 2. The optimization of daily HGF and OSM using different concentration. (a) The addition of albumin or FBS were essential to preserve the degradation of HGF and OSM in cell free condition. (b) The optimization of the HGF and OSM concentration in the last stage of differentiation in D/HD. (n=4 biologically independent experiments). Statistical significance: *p < 0.05; **p < 0.01; ***p < 0.001; ****p < 0.0001.

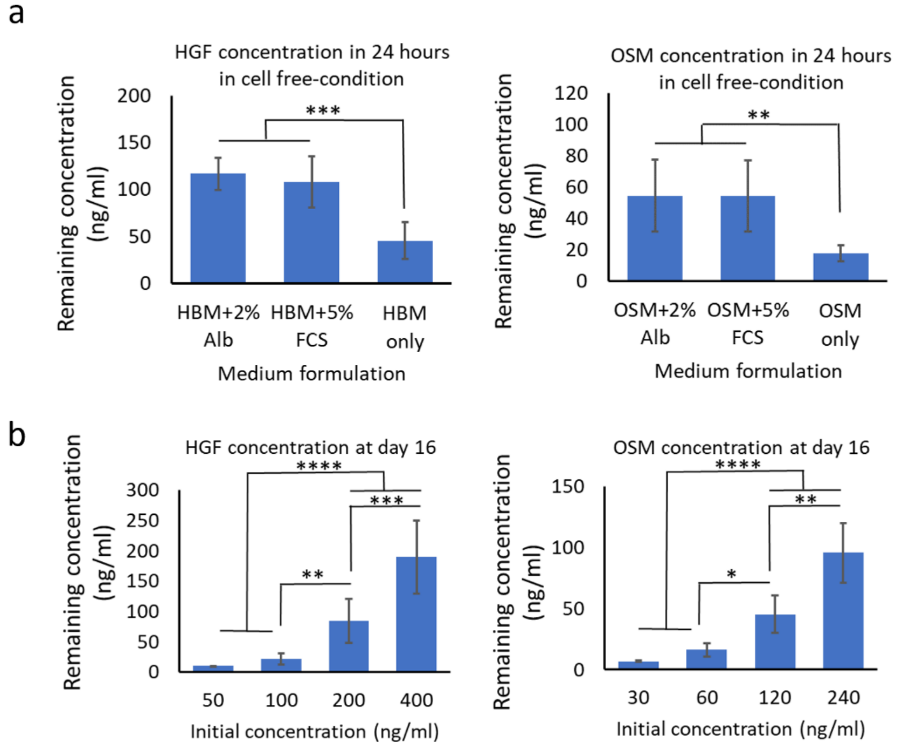

Supplementary figure 3. Approximate cost production of hepatic cells using each culture configuration. The D/HD showed significantly less exogenous growth factors requirement per million cells during HLOs production (a). This high efficiency in D/HD impacting its cost savings by improving cost production efficiency. This configuration becoming the cheapest HLOs generation strategy among the culture system, while forcing the high-density culture in conventional medium replacement system (C/HD) showed a slightly higher cost production than performing differentiation in the same culture system using low cell density (C/LD) (b). The calculation was based on exogenous growth factors and basal medium usage and their price in September 2021 (n = 4 biologically independent experiments). Mean ± standard deviation is indicated in each graph. Statistical significance: **p < 0.01; ns: not significant.

a

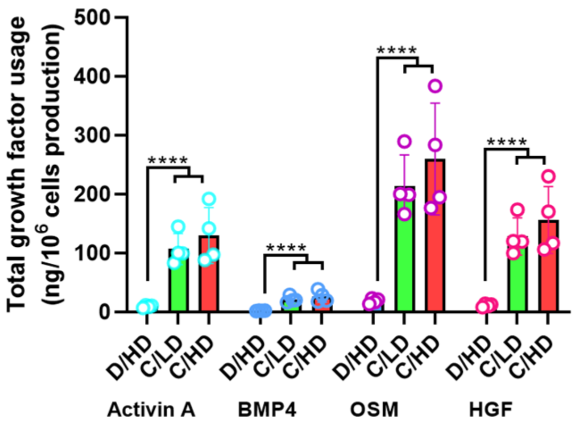

b

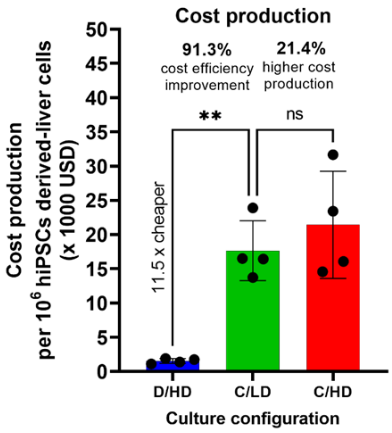

Supplementary table 1. The primer sequence used in this study.

Gene target	Forward primer	Reverse primer
CYP3A4	ACATAGCCCAGCAAAGAGCAAC	GTCTGGGATGAGAGCCATCACT
ALB	CCTGCTGACTTGCCTTCATTAG	TGGCATAGCATTCATGAGGA
KRT19	GCCACTACTACACGACCATCCA	AGAGCCTGTTCCGTCTCAAACT

Supplementary table 2. The antibodies used for ELISA and immunocytochemistry analysis

Antibodies / kit	Analysis	Information	
		Catalog no.	Manufacturer
Sheep Anti human Cytokeratin 19	Immunocytochemistry	AF3506SP	R&D Biosystems
Goat Anti human Albumin	Immunocytochemistry	A80-129A	Bethyl
Rat anti-human Cytokeratin 7 Antibody, Alexa Fluor® 647 conjugated	Immunocytochemistry	601607	Biolegend
Rabbit Anti-Sheep IgG Alexa Fluor® 555	Immunocytochemistry	ab150182	Abcam
Donkey Anti-Goat IgG Alexa Fluor® 488	Immunocytochemistry	ab150129	Abcam
DAPI	Immunocytochemistry	340-07971	Dojindo
Human HGF Quantikine	ELISA	DHG00B	R&D Biosystems
Human OSM DuoSet	ELISA	DY295	R&D Biosystems
Human Activin A DuoSet	ELISA	DY338	R&D Biosystems
Human BMP4 DuoSet	ELISA	DY314	R&D Biosystems
